# Job Demands, Resources, and Future Considerations: Academics' Experiences of Working From Home During the Coronavirus Disease 2019 (COVID-19) Pandemic

**DOI:** 10.3389/fpsyg.2022.908640

**Published:** 2022-06-21

**Authors:** Işıl Karatuna, Sandra Jönsson, Tuija Muhonen

**Affiliations:** ^1^Department of Psychology, Faculty of Social Sciences, Beykoz University, Istanbul, Turkey; ^2^Department of Urban Studies, Centre for Work Life and Evaluation Studies (CTA), Malmö University, Malmö, Sweden; ^3^Department of School Development and Leadership, Centre for Work Life and Evaluation Studies (CTA), Malmö University, Malmö, Sweden

**Keywords:** COVID-19, higher education, academics, job demands and resources, qualitative

## Abstract

The coronavirus disease 2019 pandemic has severely affected workers, workplaces, and working practices. In the higher education setting, universities have shifted to distance learning, resulting in profound changes in academics' work. In this study, we aimed to describe academics' job demands and resources related to changes in working conditions during the pandemic, and to examine how these changes have affected the perceived occupational wellbeing of academics. Additionally, we aimed to investigate academics' expectations and concerns for future academic working practices following the pandemic. The data were collected through semi-structured interviews with 26 academics working at various universities in Sweden. A content analysis was used to identify the key themes from the transcribed interviews. The results indicated that academics experienced a lack of face-to-face communication, absence of an academic environment, work overload, and work-home interference as demanding during the pandemic. In relation to resources, online communication options, appropriate working conditions, organizational-social support, and individual factors were perceived as important. Most respondents perceived negative occupational wellbeing outcomes. However, academics who had the appropriate resources were less likely to be affected by job demands. Academics' expectations for future academic work included continuation of working online, flexibility in the choice of workspace, and strengthened digital capacity. Their concerns were related to a lack of face-to-face interaction, management actions and economic implications, and pure digital education. This paper contributes to the literature by illustrating the complexity and diversity of experiences and preferences among academics that are important for universities to consider when organizing and managing future academic work.

## Introduction

The unprecedented global coronavirus disease 2019 (COVID-19) pandemic has severely affected workers, workplaces, and working practices. In the higher education setting, universities have shifted to distance learning and academics have had to teach using digital interfaces, hold online meetings and webinars instead of on-campus events, and attend virtual conferences and symposia because of the cancellation or postponement of face-to-face gatherings (Marinoni et al., 2020). Consequently, academics have had to rapidly learn how to manage their work under substantially changed conditions (Sjølie et al., 2020).

These changes have led to increased job demands and diminished resources for many academics (de Rijk, 2020). A combination of increased job demands and diminished resources can have serious consequences for academics, including increased stress levels and reduced wellbeing (Bakker and Demerouti, 2017, 2018). In the current study, we aimed to describe academics' job demands and resources related to changes in working conditions during the COVID-19 pandemic, and to examine how these changes have affected the perceived occupational wellbeing of academics. Perceived occupational wellbeing is a type of work-related wellbeing that is defined as the “evaluation of various aspects of one's job, including affective, motivational, behavioral, cognitive and psychosomatic dimensions” (van Horn et al., 2004, p. 366–377).

In the current study, we also examined the expectations and concerns about academic working practices following the end of the pandemic, because we believe that different types of digitalized working methods and hybrid solutions will characterize new academic working practices in the future.

### Theoretical Perspective

We used the job demands and resources (JD-R) model to examine academics' perceptions of changes in working conditions and their occupational wellbeing during the COVID-19 pandemic. According to this model, every occupation has unique work characteristics that affect employee wellbeing and that can be classified into two broad categories: job demands and job resources (Demerouti et al., 2001). Job demands are aspects of jobs that require sustained effort and thus are associated with physiological and psychological costs. Job resources are aspects of jobs that function to achieve work goals, stimulate personal growth, and to reduce job demands and their associated physiological and psychological costs (Demerouti et al., 2001). Personal resources (i.e., individuals' sense of their ability to successfully control and have an impact on their environment; Hobfoll et al., 2003) play a similar role to that of job resources in the JD-R model (Bakker and Demerouti, 2018).

The JD-R model posits that job demands (e.g., work pressure, work overload) lead to impaired health and exhaustion, whereas job resources (e.g., autonomy, sense of belonging) and personal resources (e.g., self-efficacy, optimism) promote work-related motivation and work engagement (Bakker and Demerouti, 2007; Schaufeli and Taris, 2014). Furthermore, the model suggests that adequate resources buffer the negative effects of increased job demands (Bakker et al., 2005).

Previous research in higher education has used the JD-R model to conceptualize the role of job demands and resources in academia (Bakker et al., 2005, 2010; Rothmann and Jordaan, 2006; Boyd et al., 2011; Barkhuizen et al., 2014; Mudrak et al., 2018; Nurendra, 2018; Converso et al., 2019; Han et al., 2020; Naidoo-Chetty and du Plessis, 2021a; Holliman et al., 2022). Some of the resources identified are organizational support, trust in the head of department/senior management, job security, social support, role clarity, meaningful work, reward, teacher efficacy, and workplace autonomy. Job demands include factors such as work overload, work pressure, conflict with colleagues, online teaching, complexity of student support, and work–home interference. More recently, in a review study on academics' job demands and resources, Naidoo-Chetty and du Plessis (2021b) reported that academic employees experience more quantitative job demands (e.g., workload, work pressure) than qualitative job demands (e.g., work-home interference, emotional demands).

In addition to the demands mentioned above, COVID-19 has placed additional burden on academics and caused imbalances between their job demands and resources (de Rijk, 2020). For example, the pandemic has created substantial challenges associated with the sudden switch to working from home and the transition to online teaching, learning, mentoring, and research activities. These considerable changes have led to increased job demands (e.g., learning new online skills under time pressure, blurring of the boundaries between work and private spaces) and diminished resources (e.g., lack of feeling connected) for many academics (de Rijk, 2020).

In line with the JD-R model and the previous empirical research described above, a combination of increased job demands and lack of resources could thus lead to increased stress levels and thereby reduced wellbeing among academics during the COVID-19 pandemic. In contrast, having adequate resources would make it easier for academics to cope with their job demands, resulting in increased wellbeing during the COVID-19 pandemic. Accordingly, academics with sufficient resources may have positive perceptions of the changes to working conditions during the pandemic. For example, a recent study showed that despite the increase in job demands, academics' job satisfaction and work engagement remained high, particularly if their workplaces provided job resources such as a positive social climate or a high level of influence over their work (Mudrak et al., 2018).

### The Rationale of the Present Study

Recently, a growing body of research has begun to elucidate the experiences of academic staff while working from home during the COVID-19 pandemic. These studies have reported that some academics perceive the shift to home and online working as an unwelcome experience characterized by stressors such as social isolation and a rapid shift to online education (Leal Filho et al., 2021; Watermeyer et al., 2021). However, other studies found that academics reported that working from home during the pandemic has provided more flexibility and more time to concentrate on their work (Esteves et al., 2020). Furthermore, although working from home has been disempowering, demoralizing, and dehumanizing for some academics, it has been empowering, agentic, and humanizing for others (Variyan and Reimer, 2021).

Academics' varying responses to changing working conditions can potentially be explained by the job demands and resources they experience during the pandemic. Several quantitative studies have provided valuable insights into academics' experiences of job demands and resources during the pandemic (Kulikowski et al., 2021; Mäkiniemi et al., 2021; Wood et al., 2021; Garraio et al., 2022; Ghislieri et al., 2022; Taylor and Frechette, 2022). However, to the best of our knowledge, there is a lack of qualitative studies in this area (Naidoo-Chetty and du Plessis, 2021a). Thus, we aimed to contribute to this emerging field of research by conducting a descriptive qualitative study that explores academics' perceptions of job demands and resources related to changes in working conditions in academia during COVID-19, and examines their perceptions of how these changes have affected their occupational wellbeing. Additionally, we aimed to investigate academics' expectations and concerns about future academic working practices following the end of the pandemic.

Therefore, the following research questions were proposed:

- How do academics perceive their job demands and resources during the COVID-19 pandemic?
- What are academics' perceptions regarding the ways that the changing working conditions have affected their occupational wellbeing?
- What are academics' expectations and concerns related to future academic working practices once the pandemic is over?

## Materials and Methods

A descriptive, qualitative design was used to gain an in-depth understanding of academics' experiences of working during the COVID-19 pandemic.

### Participants and Procedure

We interviewed academics working at various universities in Sweden. The inclusion criteria for participation in the study were as follows: performing teaching and/or research duties, speaking English, and being willing to participate. Therefore, using a purposive sampling method, participants were initially recruited *via* an announcement and invitation to participate in the study placed in a staff newsletter at a university in the south of Sweden. We then emailed the announcement to academics at different Swedish universities. In addition, we used snowball sampling recruitment (Noy, 2008), in which previously interviewed participants nominated other potential participants. Using the snowball sampling method in addition to the purposive sampling method offered the opportunity to increase the sample size of the study (Ritchie et al., 2003). Sampling continued until data saturation was reached (i.e., the point at which collected data no longer brought up anything new) (Schreier, 2012). Overall, 26 university academics from five Swedish universities participated in the study.

Participants comprised 19 senior lecturers, one lecturer, two professors, and four doctoral students. Participants were aged between 33 and 67 years (mean age: 50 years). Of the 26 participants, nine were women and 17 were men (see Table 1).

**Table 1:** Participant characteristics.

**ID**	**Position**	**Gender^a^**	**Age**	**Years of experience in academia**
P1	Senior lecturer	M	51	23
P2	Senior lecturer	F	58	13
P3	Senior lecturer	M	55	17
P4	Senior lecturer	M	54	25
P5	PhD student	F	44	4
P6	Senior lecturer	M	57	6
P7	PhD student	F	33	4
P8	Senior lecturer	M	50	18
P9	Senior lecturer	F	64	23
P10	Lecturer	F	45	19
P11	Senior lecturer	M	48	7
P12	Professor	M	67	22
P13	Professor	F	65	15
P14	Senior lecturer	M	62	10
P15	Senior lecturer	M	54	3
P16	Senior lecturer	M	40	10
P17	Senior lecturer	M	38	6
P18	Senior lecturer	M	50	10
P19	Senior lecturer	F	48	4
P20	Senior lecturer	F	42	18
P21	Senior lecturer	M	41	16
P22	PhD student	M	34	6
P23	PhD student	M	46	11
P24	Senior lecturer	M	54	23
P25	Senior lecturer	M	50	13
P26	Senior lecturer	F	51	12

a *F, female; m, male*.

The data were collected using semi-structured interviews. All interviews were conducted in English by the first author between October 2020 and January 2021. The author who conducted the interviews had no prior established relationships with the interviewees. The interviews lasted between 30 and 60 min and were conducted *via* Zoom, except for one face-to-face interview. Participants were interviewed using a semi-structured interview guide, which was developed by the researchers based on the study objectives and a review of the literature. The interview guide consisted of open-ended questions that helped to frame the interviews and permitted probing for additional information (Miles and Huberman, 1994).

Participants were initially asked to describe the main changes in their working conditions during the COVID-19 pandemic, and the perceived difficulties (job demands) and positive factors (job/personal resources) associated with these changes. They were then asked to explain whether they perceived that these changes had affected their occupational wellbeing. Finally, they were asked to express their concerns and expectations about future academic working practices following the end of the pandemic.

The interviews were audio-recorded. Before audio-recording, informed consent was obtained from all participants, including consent for digital recording. The study was approved by the Swedish Ethical Review Authority (Ref nr. 2020-04704).

### Data Analysis

The recorded narratives were transcribed verbatim, and the data were analyzed using qualitative content analysis (Krippendorf, 2004; Schreier, 2012). The verbatim transcripts of academics' working experiences during the COVID-19 pandemic constituted the unit of analysis. We chose to use qualitative content analysis since it is an appropriate and commonly used method for analyzing qualitative data (Hsieh and Shannon, 2005). Additionally, it enables the researchers to apply both inductive and deductive approach for analyzing the research data (Vaismoradi et al., 2013). Therefore, in the present study, we generated an initial coding frame deductively based on previous studies that had used the JD-R model in the context of research on higher education institutions (Bakker et al., 2005, 2010; Rothmann and Jordaan, 2006; Boyd et al., 2011; Barkhuizen et al., 2014; Naidoo-Chetty and du Plessis, 2021a,b). Because little is known about how the COVID-19 pandemic has affected academics' working experiences, the coding frame also remained open to any inductive codes. Thus, using a data-driven perspective, each author read through three interview texts and made notes on potential additional codes and categories. Thereafter, all of the authors discussed the initial codes and a coding frame was developed (see Table 2). The first author then applied the developed codes to subsequent interview texts. The results of the analyses were discussed and revised when necessary, to achieve interpretive consensus (Patton, 1987).

**Table 2:** Themes categories and example codes, including the number of participants mentioning each theme.

**Themes**	**Categories**	**Example codes for each category**	**Number of participants mentioning each theme *n* (%)**
Job demands	Lack of face-to-face communication	Lack of face-to-face communication with students/ colleagues, communication barriers in online teaching	26 (100)
	Absence of academic environment	Miss discussions with colleagues in person, lack of networking in conferences/seminars	19 (73)
	Work overload	Back-to-back zoom meetings, learn new teaching techniques in a short time	17 (65)
	Work-home interference	Lack of appropriate working space, blurred boundaries between work and private life	7 (26)
Job and personal resources	Online communication options	Easy attendance to different work-related activities, virtual hangouts with colleagues	19 (73)
	Appropriate working conditions	Having adequate space at home or outside, good internet connection	18 (69)
	Organizational and social support	Support from IT department/colleagues/manager, having someone to talk to at home	15 (57)
	Individual factors	Being experienced in online teaching, good phase in research activities	8 (30)
Occupational wellbeing	Negative outcomes	Decreased motivation, feeling tired of long zoom days, feeling lonely, decreased work engagement	23 (88)
	Positive outcomes	Healthier lifestyle, efficient working, developed digital skills	11 (42)
Expectations for future academic work	Continuation of working online	Continue workplace meetings by zoom, partial digitalisation of teaching	21 (80)
	Flexibility in the choice of work space	Option to choose between working from home and working at university, increased tolerance of not being in place	11 (42)
	Strengthened digital capacity/skills	Good digital capacity, universities that enhance home-based work environment	7 (26)
	Opportunities for physical meetings	Smaller offices for concentrated collaborative work, coffee rooms where students and teachers come together	6 (23)
Concerns for future working arrangements	Lack of face-to-face interaction	Not meeting colleagues/students in person, falling apart of social groups	25 (96)
	Management actions and economic implications	Less academic freedom, less space at university	9 (34)
	Pure digital education and hybrid solutions	Teaching all courses through zoom, pure digital meetings better than hybrid ones	7 (26)

### Ensuring Trustworthiness

To establish the trustworthiness of the study, a member check process (Lincoln and Guba, 1985) was used immediately after transcribing the recorded interviews. Transcribed interviews were shared with each participant to confirm their statements, and participants' suggestions were considered during the data analysis process. Transferability was addressed by considering the characteristics of the participants and describing the study procedure in detail. Furthermore, we used direct quotations when presenting data findings to ensure credibility (Lincoln and Guba, 1985).

## Results

The JD-R model was used as an analytical framework. The analysis identified five overall themes: job demands, job and personal resources, occupational wellbeing, expectations about future academic working practices, and concerns about future working practices. Different categories emerged that were related to the five overall themes (see Table 2).

### Job Demands

This theme represents academics' experiences of the job demands that emerged during the pandemic and comprised four categories: “lack of face-to-face communication,” “absence of academic environment,” “work overload,” and “work–home interference.”

#### Lack of Face-to-Face Communication

The most frequently reported job demand was the lack of face-to-face communication. Many academics mentioned the difficulties they experienced because of a lack of face-to-face interaction with students, and how communication barriers in online teaching had made it difficult to perform their work.

“*I think it's a bit more tedious when you supervise students… to give appropriate feedback… it's normally better if you sit around the table and have a discussion… you get a better flow of social dynamics*.” (P11)

Teachers who were involved in courses that require a lot of practical work and one-on-one teaching (e.g., design courses), struggled with the digital teaching format. Many aspects of the learning environment for these types of courses, such as being able to see and feel the materials, collaboration between students, and creative discussions, were difficult to achieve in a digital setting.

“*I'm teaching design. Design is hands on. Our curriculum is about studio work. That inherently has to be live… You can always have elements that are online, but what is the novelty in that? The things that really matter are being there, playing with materials, being inspired by others, meeting and speaking in the breaks*.” (P6)

In addition to the lack of communication with students, almost all academics complained about difficulties experienced because of a lack of communication with colleagues.

“*With the Zoom meetings, you don't get any natural breaks in which you can go and get a cup of coffee and have social conversations. You just focus on the topic and don't socialize much… I miss that*.” (P13)

Some participants also mentioned how they had to put more effort and time into communicating with others. Other difficulties arising from a lack of face-to-face communication were related to reduced contact with less close colleagues, acting as a leader, conducting hybrid lectures/meetings, and conducting online examinations.

#### Absence of Academic Environment

Participants reported that they missed the academic environment, discussions with colleagues, work-related travel, and networking at physical seminars and conferences. Online seminars and conferences did not offer the same spontaneous possibilities for discussion and exchange of ideas.

“*You get a lot of new ideas just going to a conference… Even though you meet people online it's not the same thing. I mean, just going to a conference and chatting with new people… I haven't found a good way to do that online*.” (P16)

Regarding research, many academics reported that they experienced difficulties in satisfactorily conducting research. The most frequently mentioned problems were disrupted communication with research groups, postponement or cancellation of research projects, and difficulties collecting data.

#### Work Overload

Many academics reported that their workload had substantially increased during the pandemic (e.g., owing to increases in working hours, number of emails, and administrative tasks). However, the most frequently mentioned factor that increased academics' workload was spending a lot of time in online meetings.

“*It takes a lot of time with all these meetings… When I worked at home before, I could find time to work on my own stuff, and now I can't… because there are lots of meetings all the time*.” (P9)

Participants also found it taxing to conduct large online meetings or teach large classes because of the impossibility of monitoring and directly communicating with every individual. They felt that communication *via* online chat did not help to build dynamic relationships.

“*I was teaching a class last week… there were 68 participants. It is extremely difficult to have an overview of 68 persons… This does not allow you to build dynamic relationships*.” (P1)

Academics felt that rapid digitalization had increased their workload. They had had to learn new teaching techniques and adapt courses to digital format in a very short period. Another demand that increased workloads was the need to put more energy into planning teaching and making lectures interesting.

“*I have to plan things in a different way; I have to be more prepared for what I am going to do next Tuesday… that's quite difficult*.” (P3)

#### Work–Home Interference

An additional job demand that was mentioned was work–home interference. Lack of appropriate working space, lack of distance between work and personal space, having children at home, and blurred boundaries between work and private life were difficulties experienced related to work–home interference during the pandemic.

“*I'm in the living room now. I worked in the kitchen from the morning until just half an hour ago. I will probably be upstairs in the afternoon because two of my younger children are at home and my wife is working from home. She has meetings all day long. She has to be in the study. So, I've been pushed around*.” (P18)

### Job and Personal Resources

This theme reflected academics' perceived resources during the pandemic, and comprised four categories: “online communication options,” “appropriate working conditions,” “organizational and social support,” and “individual factors.”

#### Online Communication Options

Some participants highlighted the importance of regular online communication in the workplace. This was achieved by regular departmental meetings organized by managers, but also by social gatherings and daily coffee meetings.

“*Our boss… she has been very good at keeping up this structure of Monday morning meetings and meetings on both Wednesdays and Fridays; we have online social gatherings… And now some of us have signed up for some health challenge groups, where we're going to do sports on our own and then be part of a team*.” (P7)

However, participants felt that these types of gatherings should be optional because not everyone wanted to participate in them. The most important thing was to find ways to communicate with colleagues during the workday.

Many participants also mentioned how virtual communication made it easier to attend different work-related activities and to extend invitations to guest researchers/teachers, and how workplace meetings were shorter and more efficient. Some also felt that online meetings created equal opportunities for individuals.

“*I have students now from Sri Lanka and Ireland… so that's a lot of time zones… And they shouldn't be discriminated just for not being able to be here now. They should have the same chance to contribute, to interact in seminars, as students that have the opportunity to be here.”* (P17)

Another job resource related to working online was the freedom to organize work in a more flexible way.

“*It's different working in this way, but I wouldn't say it's bad. I like the freedom… I can adjust a lot of my work tasks, so it suits me*.” (P22)

Some academics who commuted before the pandemic stated that the new flexible working conditions permitted by online working ensured more humane and efficient working hours.

#### Appropriate Working Conditions

Having appropriate working conditions helped many academics to perform their work during the pandemic. For example, academics who had adequate equipment, a good internet connection, and a private work area either at home or outside (e.g., a private office) were more likely to report positive experiences regarding their working conditions during the pandemic.

“*I have my own workroom… I have had that before too, a really good work environment*.” (P13)

In addition to a good physical workspace, participants placed importance on the social aspect of work. For example, having routines and somebody to talk to during the work day were considered important resources.

“*I have my husband here. I think it's quite good… we get up, we have breakfast, and then we each go to our own rooms to have our meetings and do our work, and then we meet for lunch breaks … so we have become colleagues in some ways… So, I think it's quite an advantage… It would be much lonelier if I was sitting here alone*.” (P2)

#### Organizational and Social Support

Participants appreciated the support they had received during the pandemic period from the information technology and administrative departments and from their managers.

“*I am getting support from my faculty for sure. Our head of department is wonderful; she has been meeting with us continuously during this period and has definitely been trying to support us.”* (P5)

Furthermore, support from colleagues was identified as an important resource that helped participants adapt to their new job demands.

“*We get a lot of support … we are using Slack as the main communication tool, and we use it a lot… So, I think that I meet my boss more now than I did before … I have more communication with him now than I did before. So, in one sense, that's a positive thing*.” (P16)

#### Individual Factors

Individual strengths or resources, such as being disciplined about work organization and being positive about completing tasks, were identified as personal resources that helped academics to better manage their work during pandemic conditions.

“*I don't have a big problem in sort of motivating myself… I just sit down and work. It works for me*.” (P21)

Some participants had previous experience with online teaching, which made the transition easier and less problematic.

“*Most of the teaching I've been doing… it has worked out pretty well in an online format… We usually publish all our lectures online… So that is not a major challenge for us*.” (P16)

In relation to the research process, some academics experienced no problems with their research because they did not need to conduct empirical work. Others had completed field work and were focusing on data analysis, and therefore felt that their research was not negatively affected during the pandemic.

“*I think I got lucky there, because I don't have any empirical work to carry out, so I wasn't forced to cancel anything. Right now, my work is theoretical, so I did not experience any difficulties*.” (P3)

## Occupational Wellbeing

This theme describes academics' perceptions of occupational wellbeing outcomes of working during the COVID-19 pandemic and comprised two categories: “negative outcomes” and “positive outcomes.”

### Negative Outcomes

Most academics emphasized the negative wellbeing outcomes they had experienced during the pandemic. Almost all participants felt that the lack of face-to-face communication with others had led to negative outcomes such as feeling lonely, feeling left out, missing friends and colleagues, social incompetency, and social starvation.

“*I've got no oxygen… I don't get the kind of feedback that keeps me alive and kicking. It's not a lively work environment anymore—that's the oxygen, so to speak. I just feel like a fish out of water… still wriggling*.” (P6)

Some participants reported how lack of face-to-face communication with students and colleagues had challenged their ability to stay engaged with their profession and had made them rethink their career.

“*If this is going to be the norm, sitting alone at home… not meeting students… No, then I would probably find a new job. I'm not suited to this*.” (P1)

Furthermore, some academics reported exhaustion and physical health complaints, such as feeling tired and less energetic. Reduced motivation and performance were also mentioned as negative occupational outcomes of the job demands experienced during the pandemic.

“*I think my performance has also been affected to some extent. I mean, if you think student-wise, I think it's difficult to be at the top of your game when you're not in your classroom.”* (P11)

Working online from home brought work into academics' private spaces (e.g., kitchen, study, living room), which are usually not shared with students and colleagues. Blurred boundaries between private and work life could result in negative feelings, such as loss of a sense of freedom, and the feeling of not having done enough, despite working all day.

“*It's just like I'm working all the time; I never feel free, and I never feel that I've done enough when I work at home*.” (P19)

Some academics complained of feeling less professional than before, of losing the feeling of being part of an academic team, and of how their academic development had been negatively affected by online working from home. Other negative outcomes participants experienced during the pandemic were reduced inspiration, morale, and work discipline, and increased stress, boredom, and hopelessness.

### Positive Outcomes

Although most academics mentioned negative wellbeing outcomes, some emphasized the positive outcomes of working during the pandemic.

“*I think it is a healthier lifestyle, not being at work as much… being at home more. But there should be a balance. You need to see some colleagues as well*.” (P2)

Some mentioned how working from home allowed them more time for themselves. In addition, some participants liked spending less time commuting and having more time to focus on their work.

“*It is quite convenient not to spend 3 hours commuting. It gives you more freedom to spend time walking in the forest and being with your family… I can see that I can actually accomplish more when I work from home… I think it is a healthier lifestyle, spending less time at work and more time at home.”* (P2).

Some participants felt good about the new working conditions. Perceptions of positive wellbeing outcomes included more work efficiency, the development of digital skills, and feeling more prepared to engage with new digital tools.

“*I think it's fun to try new technologies. I'm very fond of trying new things. So, for me, this has been like a very fun journey where I can try new tools. I've learned to use Open Broadcaster Software Studio. I've learned a lot about YouTube streaming, Zoom, Teams, and all that. And I think it's super fun.”* (P16)

The aspect of effectiveness was also mentioned in relation to digital teaching and supervision.

“*Teaching through digital devices… It's very time effective… to manage supervision and things like that.”* (P24)

## Expectations About Future Academic Working Practices

This theme encompassed academics' expectations about future academic working practices and comprised four categories: “continuation of working online,” “flexibility in the choice of workspace,” “better digital resources/skills,” and “opportunities for physical meetings.”

### Continuation of Working Online

Many academics reported that they would like to continue having workplace meetings *via* Zoom once the pandemic was over.

“*Workplace meetings have gone so smoothly; it would be interesting to have them [online] in the future*.” (P22)

Some also mentioned that continuation of online activities would reduce spending on office space, thus enabling more money to be spent on students. Similarly, some participants highlighted how continuation of digital meetings and conferences would reduce traveling and commuting time, thus helping to protect the environment.

“*Cutting this mobility … would have positive consequences for the environment and so on… we should restrict our mobility in that sense … that could be a positive aspect*.” (P12)

Academics' other expectations about future academic working practices included virtual options for conferences, partial digitalization of teaching activities, online workshops/seminars with fewer people, and keeping some parts of teaching online.

### Flexibility in the Choice of Workspace

Although some academics expected a return to more on-campus work, others stated that they would like to work more from home, and many wished to retain the option to choose between workspaces following the pandemic. Another expectation was that there would be greater tolerance toward not being on campus following the pandemic.

“*You should be at work… That was the norm before COVID-19… I mean, you were supposed to be at work because being a good colleague was being at work… I hope that this norm has changed now*.” (P16)

### Better Digital Resources/Skills

Frequently reported expectations about future academic work were greater digitalization, improved digital skills, better virtual communication resources, and more possibilities for hybrid work.

“*We do need to have good virtual resources to be able to allow people to participate via Zoom, but also accommodate people attending the seminar in person. So, there should be resources available, like a good microphone… and the kinds of things that are needed to enable both at the same time… People meeting in person but also opening it up to virtual meetings.”* (P5)

It was considered important for universities to ensure that academics have suitable equipment (e.g., to host different kinds of meetings). Furthermore, participants reported that it was important for universities to enhance the home-based work environment.

“*I think it is very important to take care of the home environment; we should do that in the long run… provide furniture for teachers. We have brought this issue up in the university… I think we should make some kind of contribution if people want to invest in their home environment*.” (P24)

### Opportunities for Physical Meetings

Participants also emphasized the importance of being able to have physical meetings once the pandemic was over. Accordingly, they anticipated having smaller offices for concentrated collaborative work, university hubs in different cities for commuters, and coffee rooms where students and teachers could come together.

“*I've heard people talking about hubs, that the university is creating hubs in difference places outside the campus. And I thought that was a wonderful idea. It will give me the option to go to these facilities and meet the colleagues that I would usually meet on the train.”* (P4)

Some participants also felt it was important to consider the design of office space once academics returned to work on campus.

“*My dream scenario would be to maybe have some smaller office environments… to have a more concentrated collaborative base… I think that is a very good, focused way of working.”* (P2).

## Concerns About Future Academic Working Practices

This theme encompasses academics' concerns about future working conditions and comprised three categories: “lack of face-to-face interaction,” “management actions and economic implications,” and “pure digital education and hybrid solutions.”

### Lack of Face-to-Face Interaction

The most frequently reported concern about future work in academia was the lack of face-to-face interactions with others. Almost every participant talked about their concerns about not meeting colleagues in person, not having physical meetings with students, and not experiencing informal networking at conferences in the future.

“*Meetings where you interact, you catch up, see the glow in the eye of your colleague when you develop an idea, talk to people over coffee… meet a few people you may not have met before: that doesn't happen on Zoom or any other platform.”* (P6)

Face-to-face meetings with colleagues were also described as important for professional identity and academic organizational culture.

“*I think it's very important to meet… that's what keeps us together*.” (P24)

### Management Actions and Economic Implications

Academics talked about their concerns regarding the management of academia following the pandemic. Their major concerns were bureaucratic management, rapid management decisions, lack of decisive leadership, and management that limits academic freedom and forces academics to be on campus every day.

“*Well, when it comes to work, I can say that the worst thing would be very quick decisions from the management, like: tomorrow everything has to be digital… or… tomorrow has to be different but we don't know how… this indecisiveness*.” (P20)

“*Management that annoys us with all these details and directions and rules and meetings*.” (P8)

In addition to these concerns, some participants believed that an increase in home working would affect how universities used their physical premises. For example, if home working increased, there may be a risk of a reduction in on-campus office space.

“*Now we know that people can work from home, we might be introduced to some kind of hot desking system… And I wouldn't like that*.” (P21)

### Pure Digital Education and Hybrid Solutions

Academics described their concerns about having to teach all courses and all elements of a course online and having to move all activities (including defense thesis presentations) online.

“*We have seminars and workshops where we share experiences and so on… that's much more difficult with distance learning. It's possible, but I would say that the quality of the teaching is not as good as it usually is*.” (P14)

Some academics also reported that meetings with colleagues and students should either be physical or online, but not hybrid. These respondents mentioned the difficulty of providing equal opportunities for engagement for students who were physically present and those who were online.

“*You should have either physical meetings or distance meetings… When you have a mix, it's really difficult because the ones that are connected virtually, it's difficult to involve them 100 percent*.” (P14)

The opportunity to use online teaching when appropriate was considered a positive aspect of the changing work environment. However, participants were concerned about a future scenario in which every aspect of education was conducted online. The inability to provide students with high-quality education was considered problematic, and something that needed to be discussed within academia.

## Discussion

The aim of this study was (1) to explore academics' perceptions of job demands and resources related to changes in working conditions in academia during the COVID-19 pandemic, and to examine their perceptions of how these changes affected occupational wellbeing; and (2) to contribute to improvements in academic working practices by investigating academics' expectations and concerns for future academic working practices following the pandemic.

The qualitative content analysis identified five themes that described academics' perceptions of changes in working patterns in academia during the COVID-19 pandemic: (1) job demands, (2) job resources, (3) occupational wellbeing, (4) expectations about future academic work, and (5) concerns about future working conditions.

The most frequently reported job demand was the lack of face-to-face communication. Almost all participants indicated that this had negatively affected their occupational wellbeing. However, resources such as social support (e.g., support from colleagues) and online communication options (e.g., attending virtual social gatherings) helped many respondents to meet the social challenges of working from home during the pandemic. The importance of such resources has been highlighted in previous studies (Sjølie et al., 2020; Dinu et al., 2021). These resources are very important because they can create a feeling of togetherness among academics who work from home (Hacker et al., 2020).

Another frequently reported job demand was work overload because of the shift to online education. Recent studies have also reported that academics have experienced workload increase during the pandemic because of digitalization in higher education (Jackman et al., 2021; McGaughey et al., 2021; Watermeyer et al., 2021; Naidoo-Chetty and du Plessis, 2021a). Some of our participants reported that this had negatively affected their occupational wellbeing, but that the resource of organizational support had helped them to cope with work overload and increase their wellbeing. This finding is in line with previous findings of a substantial positive association between job satisfaction and adequate institutional resources among Australian university academics (Bentley et al., 2013).

Online communication options were another resource that academics used to manage work overload. This is not surprising, because online communication options can provide academics with more freedom to organize their work and enable more convenient access to work-related activities (Prieto et al., 2021). Furthermore, different individual factors could be considered resources for handling work overload. For example, academics who were experienced in online teaching, self-disciplined in organizing their work, or had no problems completing work tasks found it easier to respond to work overload. Similarly, in a study of Chinese employees working remotely during the COVID-19 pandemic, Wang et al. (2020) found that participants who identified themselves as more disciplined completed their work in a more efficient and timely manner.

In line with recent research (Biswakarma et al., 2021), we found that lack of physical conferences, seminars, and discussions with colleagues were perceived by academics as a job demand during the pandemic. However, resources such as online communication options compensated to some extent for the absence of the academic environment.

Consistent with findings from previous studies, work–home interference was considered another job demand among academics (Esteves et al., 2020; Naidoo-Chetty and du Plessis, 2021a). However, only a quarter of our participants perceived this job demand to negatively affect their occupational wellbeing. This may reflect the resources academics had available during the pandemic, because more than half of participants reported that having adequate space/office facilities at home or outside helped them to perform their work during the pandemic.

Consistent with our expectations (see also the description of event system theory in Morgeson et al., 2015), the novelty, disruption, and threat of the COVID-19 pandemic had a substantial effect on the perceived occupational wellbeing of academics. Most participants reported experiencing negative effects from the job demands that arose during the pandemic. Consistent with previous findings, our participants most frequently mentioned negative outcomes such as missing social contact with colleagues (Rubin et al., 2020), feeling lonely (Prieto et al., 2021), and reduced motivation (Jackman et al., 2021; Kulikowski et al., 2021). However, academics who had adequate resources were less likely to be affected by job demands; instead, they highlighted several positive aspects of working from home (e.g., healthier lifestyle, less commuting, development of digital skills).

Overall, our findings are consistent with the JD-R model (Demerouti et al., 2001). Perceived job demands during the COVID-19 pandemic led to perceptions of negative occupational wellbeing outcomes for most academics. However, perceived job and personal resources either increased academics' wellbeing or buffered the negative effects of job demands on perceived occupational wellbeing in many cases. Therefore, our findings support the assumption that the combination of high job demands, and low resources has a negative effect on perceived occupational wellbeing, and that sufficient resources can buffer this effect among academics during the COVID-19 pandemic.

The interview data demonstrated a range of responses to the issue of expectations about future academic working practices. Although most academics expected to continue using digital interaction formats following the pandemic, many felt that complete digitalization in higher education was undesirable (Eringfeld, 2021; Watermeyer et al., 2021). For instance, many academics reported that they expected to have online workplace meetings in the future. However, some respondents reported having concerns about the difficulties of participating in virtual meetings. In line with these concerns, some respondents also reported their expectations about future improvements in digital resources that would ensure the quality and efficiency of virtual lectures and meetings.

There were also conflicting expectations about how conferences would be held following the pandemic. Although academics expected to have the option of attending virtual conferences, they also emphasized the importance of networking (informal interaction) in physical meetings (Schwarz et al., 2020). Similarly, there were different expectations about the workspace; although some participants expected to continue working from home following the pandemic, others expressed a wish to spend more time on campus.

Regarding concerns about new working conditions in academia, most participants were concerned about the lack of face-to-face interaction with colleagues and students, which has also been reported in other research on academics (Eringfeld, 2021). Similarly, pure digital education and hybrid lectures were identified as important concerns that represented a risk that quality in higher education would decline. Another concern was the possibility of management actions that would threaten the flexibility and freedom academics had experienced during the pandemic. Academics who expected to have to spend time on campus in the future were also concerned about having less space or no space to work at the university.

Rather than suggesting a single approach to future working patterns in academia, the overall findings of this study indicate the need to balance the competing wishes of academics. Thus, to meet job demands in academic work following the COVID-19 pandemic, universities should increase job resources such as improving the quality of digital interaction, meeting academics' needs for physical interaction, and ensuring flexibility in the choice of workspace. At the same time, universities should enable suitable on-campus workspaces and enhance home-based work environments.

The current study had several limitations. The sample size was relatively small, and senior lecturers were over-presented. Moreover, the mean age of participants was 50 years. Thus, the current findings may have limited generalizability. Future studies should increase the sample size by including younger academics in different academic positions to obtain a deeper understanding of future academic working practices. Despite these limitations, a strength of the current study is that the results demonstrated the complexity and diversity of academics' experiences and preferences. It is important that this complexity is considered when universities organize and manage future academic work.

## Data Availability Statement

The original contributions presented in the study are included in the article/supplementary material, further inquiries can be directed to the corresponding author/s.

## Ethics Statement

The studies involving human participants were reviewed and approved by Swedish Ethical Review Authority (Ref no. 2020-04704). The participants provided their written informed consent to participate in this study.

## Author Contributions

IK, SJ, and TM contributed to the conception and design of the study and analyzed the data. IK performed the interviews, organized the data, and wrote the first draft of the paper. All authors contributed to manuscript revision, read, and approved the submitted version.

## Funding

This research was funded by Centre for Work Life and Evaluation Studies, Malmö University.

## Conflict of Interest

The authors declare that the research was conducted in the absence of any commercial or financial relationships that could be construed as a potential conflict of interest.

## Publisher's Note

All claims expressed in this article are solely those of the authors and do not necessarily represent those of their affiliated organizations, or those of the publisher, the editors and the reviewers. Any product that may be evaluated in this article, or claim that may be made by its manufacturer, is not guaranteed or endorsed by the publisher.
